# Characteristics and predictive model for diffuse large B-cell lymphoma with early chemoimmunotherapy failure

**DOI:** 10.3389/fimmu.2025.1553850

**Published:** 2025-06-16

**Authors:** Ying-Yu Dong, Qing Shi, Wen Wu, Bing-Bing Zhao, Di Fu, Peng-Peng Xu, Shu Cheng, Guilhem Bousquet, Wei-Li Zhao, Li Wang

**Affiliations:** ^1^ Shanghai Institute of Hematology, State Key Laboratory of Medical Genomics; National Research Center for Translational Medicine at Shanghai, Ruijin Hospital Affiliated to Shanghai Jiao Tong University School of Medicine, Shanghai, China; ^2^ Shanghai 411 Hospital, Shanghai, China; ^3^ Université Sorbonne Paris Nord, Villetaneuse, France; ^4^ Assistance Publique Hôpitaux de Paris, Hôpital Avicenne, Service d’Oncologie Médicale, Bobigny, France; ^5^ Pôle de Recherches Sino-Français en Science du Vivant et Génomique, Laboratory of Molecular Pathology, Shanghai, China

**Keywords:** DLBCL - diffuse large B cell lymphoma, early chemoimmunotherapy failure, RCHOP-like regimen, CAR- T cells, chemo-resistant, nomogram

## Abstract

**Introduction:**

The outcomes of refractory or relapsed diffuse large B-cell lymphoma are generally poor, especially those relapsed or progressed within 12 months from diagnosis named as early chemoimmunotherapy failure (ECF), with a 2-year OS of 24.7%. Due to the dismal outcome, early recognition of ECF and developing targeted innovative treatments to improve patient prognosis are urgent.

**Methods:**

This study recruited 2038 newly diagnosed DLBCL patients treated with R-CHOP/RminiCHOP or R-CHOP-based immunochemotherapy in Ruijin hospital and 411 hospital from December 1997 to December 2020.

**Results:**

Compared to the control group, ECF patients were significantly associated with elderly age, advanced Ann Arbor stage, elevated serum LDH, poor performance status, multiple extranodal involvements, double expressor lymphoma (DEL), and non-GCB subtype, as well as high frequencies of *TP53*, *FOXO1* and *FBXW7* mutations. Through multivariate analysis, elderly age, advanced stage, elevated serum LDH, DEL, and mutations of *TP53* or *FOXO1* were independent predictors of ECF.

**Discussion:**

Based on these predictors, a nomogram of ECF was established, and the straining cohort of our Chinese patients as well as the external cohort from Western countries showed a good predictive power of the ECF model, indicating the efficiency of our ECF predicting model, regardless of patients' race. Our ECF model allows clinicians to early recognize ECF patients, to optimize the therapeutic strategies and to improve the outcome of those chemo-resistant patients.

## Introduction

Diffuse large B-cell lymphoma (DLBCL) is the most common type of non-Hodgkin lymphoma (NHL) and represents a biologically heterogeneous entity with varied clinical and molecular features (1–3). Although 60% of DLBCL patients can be cured by rituximab, cyclophosphamide, doxorubicin, vincristine, and prednisone (R-CHOP) immunochemotherapy, the other 40% either become refractory or experience disease relapse (4, 5). The outcomes of patients with refractory or relapsed (r/r) DLBCL are generally poor. According to the SCHOLAR-1, the NCIC-CTG LY.12 and the REAL-TREND studies, refractory DLBCL has a dismal outcome, with a median overall survival (OS) of 5.9- 6.1 months (5–7). Recently, patients who relapsed or progressed within 12 months from diagnosis have been identified as early chemoimmunotherapy failure (ECF) and those who relapsed after 12 months from diagnosis are classified as late chemoimmunotherapy failure (LCF) (8, 9). Patients with ECF had a worse prognosis, with a 2-year OS of 24.7%, as compared to LCF with a 2-year OS of approximately 60-70% (10). Due to the poor outcome, early recognition of ECF and developing innovative targeted treatments to improve the outcome of ECF patients are very important. However, the clinical and molecular characteristics of ECF are still unclear and a convenient method to early identify the ECF patients is an unmet need.

There are many factors indicating or affecting the prognosis of patients with DLBCL, including the international prognostic index (IPI), cell of origin (COO), gene mutations and genetic subtypes, as well as the tumor microenvironment (TME) (11–13). IPI is a powerful tool to predict the prognosis of DLBCL patients based on five clinical characteristics including age, lactate dehydrogenase (LDH), number of extranodal involvement, Ann Arbor stage, and Eastern Cooperative Oncology Group (ECOG) performance status in the rituximab era (14, 15). According to the algorithm of Hans, DLBCL is commonly classified into germinal center B-cell-like (GCB), and non-GCB (16). During the rituximab era, the GCB subgroup showed a significantly better 3-year OS than the non-GCB subgroup (17). For the genetic landscape, the mutations of genes, such as *TP53, TBL1XR1, MYC, and FBXW7* have been reported to be unfavorable factors of DLBCL patients (18–20). Based on targeted sequencing and fluorescence *in situ* hybridization, LymphPlex classified DLBCL into seven distinct genetic subtypes with distinct prognoses, including MCD-like, BN2-like, TP53^Mut^, EZB-like, ST2-like, N1-like, and not otherwise specified (NOS), however, patients with MCD-like and TP53^Mut^ had dismal outcome (21). TME plays an essential role in DLBCL progression and chemoresistance (22). Immunosuppressive TME can promote tumor growth by recruiting immunosuppressive cells such as tumor-associated macrophages (TAMs), neutrophils, and regulatory T cells (Tregs) and accumulating exhausted T-cells (23, 24). Numerous attempts have been made to incorporate clinical and genetic markers into the prognosis prediction of DLBCL, however, a useful prediction model of ECF DLBCL is still lacking (25).

The innovations of novel immunotherapies, especially the chimeric antigen receptor-T (CAR-T) cell therapies have improved the outcome of r/r DLBCL and ECF patients (26, 27). Therefore, the prognosis of DLBCL patients may be significantly improved by early identification of ECF patients and application tailored treatment strategies, based on clinical and molecular characteristics.

In this study, we conducted genomic and transcriptomic analyses to delve deeper into the molecular and microenvironmental profiles of ECF and LCF. Additionally, we constructed a nomogram model incorporating both clinical and molecular variables to predict the risk of ECF, which might help clinicians to early identify ECF and choose tailored treatment strategies to improve patients’ prognoses.

## Materials and methods

### Patients

**Figure 1:**
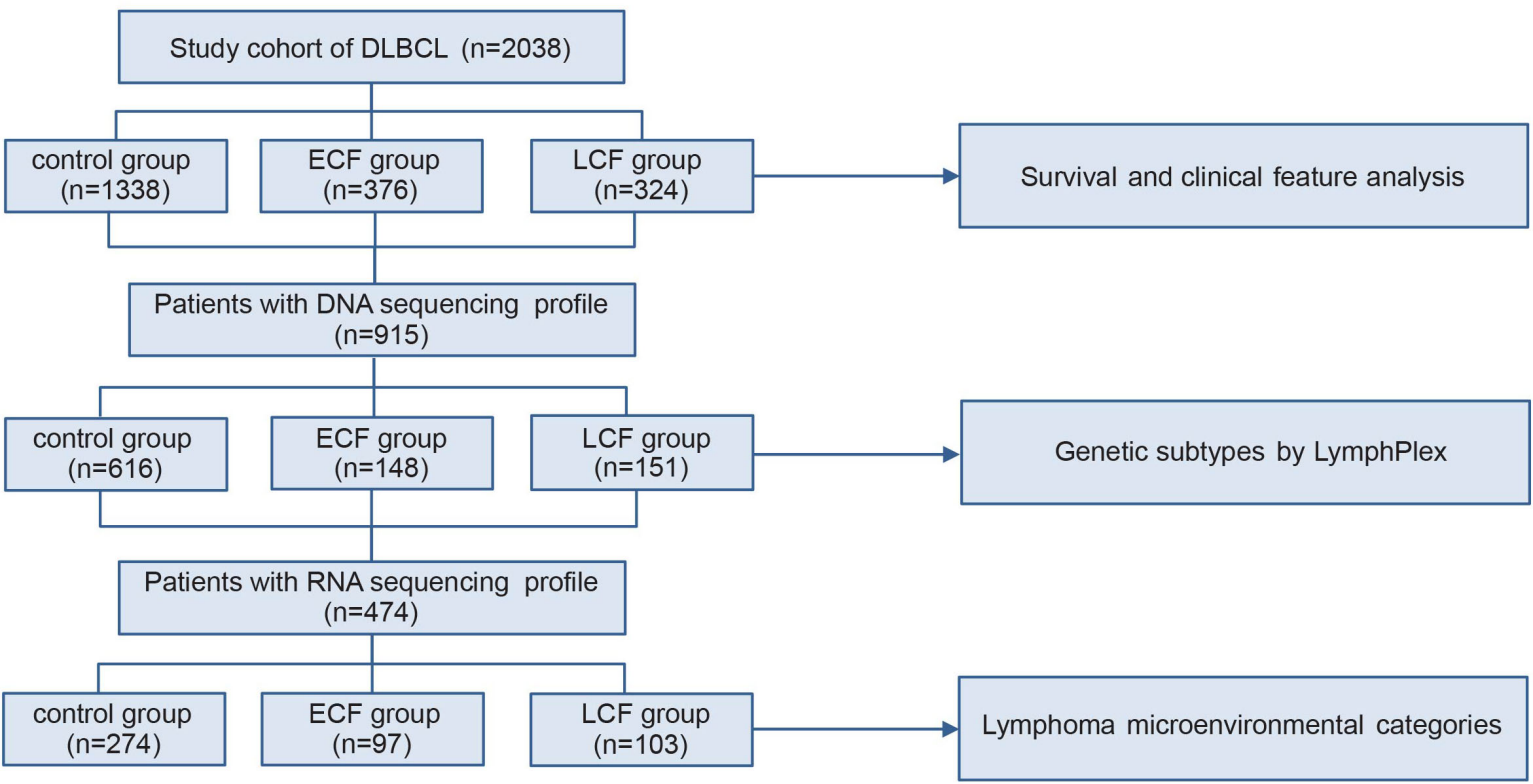
Patient flowchart. DLBCL, diffuse large B-cell lymphoma; ECF, early chemoimmunotherapy failure; LCF, late chemoimmunotherapy failure.

The selection process for patients in this study is outlined in **Figure 1**. From December 1997 to December 2020, 2038 newly diagnosed DLBCL patients (1911 from Ruijin hospital, 127 from 411 hospital) with R-CHOP/R-miniCHOP or R-CHOP-based immunochemotherapy were enrolled. Among 2038 patients, 34 elderly fit patients aged ≥ 80 years received the R-miniCHOP regimen (28) and the last follow-up was September 30, 2024. Of note, all the patients in our study received six cycles of chemotherapies. In this research, patients with central nervous lymphoma, high-grade B-cell lymphoma, and primary mediastinum large B-cell lymphoma, who underwent immunochemotherapy regimens other than R-CHOP/R-miniCHOP or R-CHOP-based therapies were excluded. This study aims to evaluate the prognosis of patients who are refractory or relapsed to chemotherapy within the context of conventional chemotherapy treatment, hence the patients who received CAR-T treatment were also excluded from this research. DLBCL patients were divided into three groups according to the initial treatment response and progression time. The evaluation time of stable disease (SD) or progressive disease (PD) was after 3–4 courses of treatment (intermediate evaluation) or at the end of treatment (final evaluation). According to SCHOLAR-1, patients with SD/PD to first-line R-CHOP regimen were identified as primary refractory patients (5). However, patients achieved complete response (CR) or partial response (PR), but progressed within 1 year from diagnosis were considered as early relapse (ER). As shown in **Supplemetary Figure 1**, patients who obtained SD/PD after R-CHOP treatment and those who relapsed within 12 months (ER) from diagnosis had similar outcome (PFS, p=0.6476 and OS, p=0.3175, **Supplementary Figure 1**). Therefore, patients had SD/PD to R-CHOP or relapsed within 12 months from diagnosis were classified as ECF, and those who relapsed or progressed after 12 months from diagnosis were classified as LCF (9). Patients who achieved CR after the first-line treatment and maintained their remission status until the last follow-up were selected as control. This study was approved by the Review Boards of both Shanghai Ruijin hospital and 411 hospital, and informed consent was obtained in accordance with the Declaration of Helsinki.

### DNA sequencing

DNA sequencing was performed on 915 patients for the detection of oncogenic mutations and genetic subtypes as previously reported (29–31). Genomic DNA was extracted from frozen tumor tissue or FFPE tumor tissue by QIAamp DNA Mini Kit (Qiagen, Hilden, Germany) and GeneRead DNA FFPE Tissue Kit (Qiagen), and whole-genome sequencing, whole-exome sequencing, and targeted sequencing covering 55 lymphoma-associated genes were performed on 67, 137, and 711 patients, respectively, as previously described (29–31). Using the GRCh37 human reference genome (version 2009-02), Samtools (version 0.1.18), Picard (version 1.93), and Genome Analysis Toolkit (version 4.1.4.0) were used for BAM file handling, local realignment, base recalibration, and calling variants, respectively. Mutations in the coding region were annotated using the Annovar software (version 2017-07-17). Variants were filtered according to the rules listed in our previous studies (32, 33).

### RNA sequencing, GSEA, and tumor microenvironmental analysis

RNA-sequencing was performed on tumor samples of 474 DLBCL patients using frozen tumor tissues. RNA was extracted using Trizol (Invitrogen, California, USA) and RNeasy MinElute Cleanup Kit (Qiagen, Dusseldorf, Germany), quantified with NanoDrop. RNA purification, reverse transcription, library construction, and sequencing were performed in WuXi NextCODE according to the manufacturer’s instructions (Illumina). The details of RNA sequencing procedures and RNA sequencing data were conducted as previously reported (29–31). Bioinformatic analyses were performed by r 4.0.3 and raw reads were normalized, and differentially expressed genes were obtained with R package “limma” (v3·38·3). Gene set enrichment analysis (GSEA) was performed with the R package “clusterProfiler” (v4.0.0) based on MSigDB-curated gene sets (c5.bp.v7.0.symbols.gmt) (34). Pathways were considered statistically significant when the P value was <0.05, and the false discovery rate was <0.25. For large-scale characterization of tumor cellular heterogeneity, cell type enrichment scores were calculated by online tools ImmuCellAI (https://guolab.wchscu.cn/ImmuCellAI), which performs cell type enrichment analysis from gene expression data for immune and stroma cell types and provides a comprehensive collection of gene expression enrichment scores for cell types (35).

### Construction and validation of the nomogram

The predictive model was constructed using clinical and DNA sequencing data from 2038 DLBCL patients. The candidate variables were screened by univariate and multivariate logistic regression analyses, followed by the development of a nomogram utilizing the “rms” package (https://github.com/harrelfe/rms). The external validation of the model was performed on the western DLBCL population from the BC Cancer (BCC) cohort (n=320) (36). In the external validation cohort, the progression-free survival (PFS) less than or equal to 12 months was considered as ECF.

### Statistical analysis

Pearson’s χ^2^ test was used to analyze the clinical and molecular characteristics of patients and LymphPlex classification across different groups. PFS was calculated from the date of diagnosis to the date of disease progression or relapse, or the date of last follow-up. OS was measured from the date of diagnosis to the date of death or the last follow-up. Survival functions were analyzed using the Kaplan–Meier method and compared by the log-rank test. Differences in gene mutations, immune cell populations and normalized gene expression in two groups were assessed using the Mann–Whitney U test. The analysis of differential genes and GSEA enrichment of gene pathways were corrected by False Discovery Rate (FDR). The Tumor mutation burden of two groups of genes was analyzed by T-test. Univariate and multivariate hazards were analyzed using the logistic regression method. All statistical analyses were performed by Statistical Package for the Social Sciences (SPSS) 26.0 software or GraphPad Prism 9. Statistical significance was defined as p <0.05.

## Results

### Clinical characteristics of ECF

**Figure 2:**
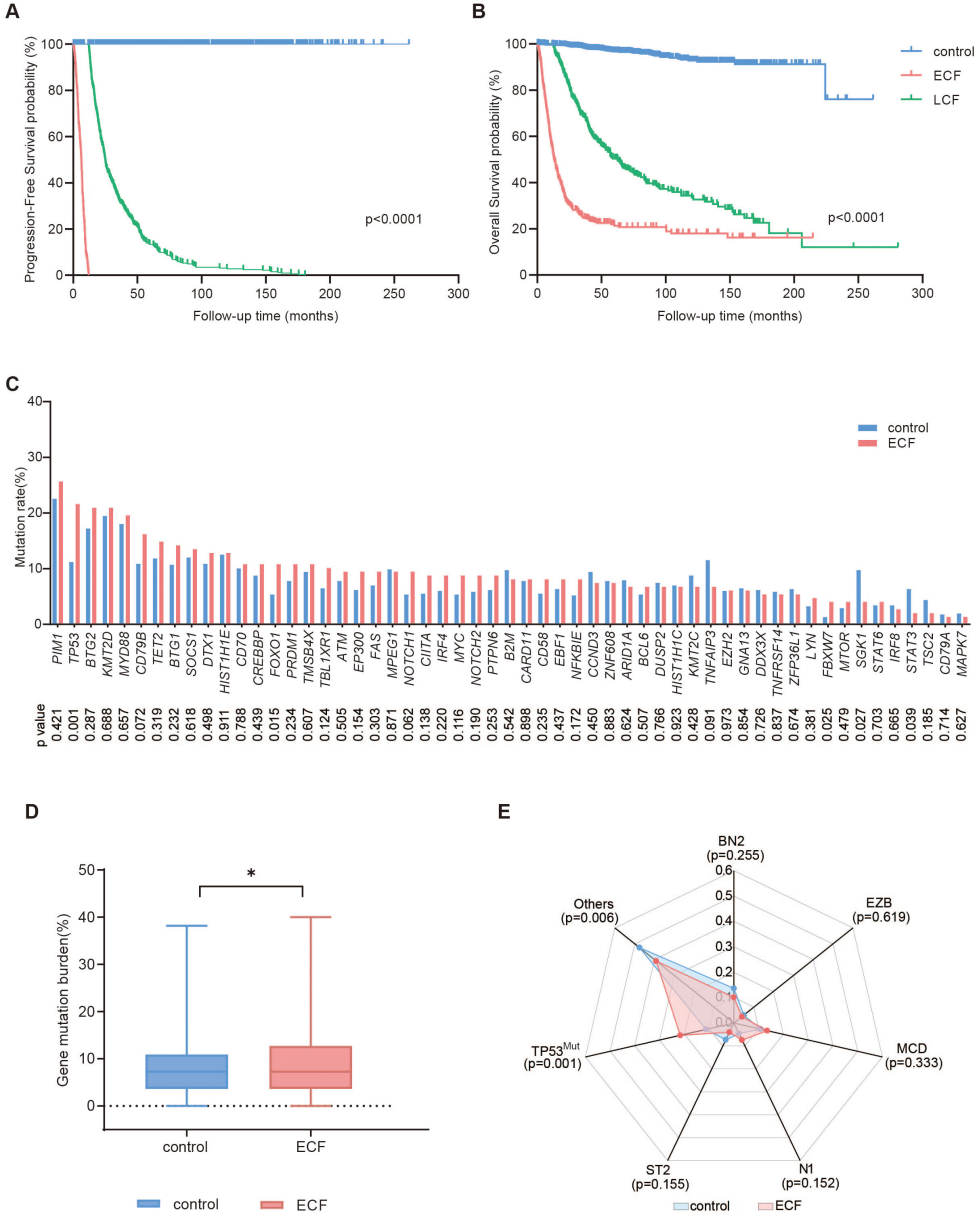
Survival and mutation analysis of DLBCL patients. **(A, B)** Progression-free survival (PFS) **(A)** and overall survival (OS) **(B)** of DLBCL patients in the control (n=1338), ECF (n=376) and LCF (n=324) groups. **(C-E)** Mutation profiles **(C)**, gene mutation burden **(D)**, and molecular subtypes **(E)** of patients in the control (n=616) and ECF (n=148) groups. The symbol * means that the comparison between the two groups is statistically different, P<0.05.

A total of 2038 patients with DLBCL were analyzed, including 1338 patients in the control group (patients without relapse or progression), 376 in the ECF group, and 324 in the LCF group (**Figure 1**). ECF patients were significantly associated with elderly age (p<0.001), advanced Ann Arbor stage (p<0.001), elevated serum LDH (p<0.001), poor performance status (37) (ECOG score more than 1, p<0.001), multiple extranodal involvements (p<0.001), DEL (p<0.001) and non-GCB subtype (p=0.022), as compared to the control group (**Table 1**). However, LCF patients were significantly associated with elderly age (p<0.001), advanced Ann Arbor stage (p<0.001), elevated serum LDH (p<0.001), multiple extranodal involvements (p<0.001), non-GCB subtype (p<0.001), poor performance status (p=0.002), and DEL (p=0.041) (**Supplementary Table 1**). In addition, when compared with LCF, ECF patients were also significantly associated with elevated serum LDH (p<0.001), poor performance status (p<0.001), advanced Ann Arbor stage (p=0.001), and DEL (p=0.036) (**Supplementary Table 2**). According to the survival analysis, the PFS of ECF patients (median PFS, 6.2 months, **Figure 2A**) was significantly shorter than those in the control group (median PFS, unreached, p<0.001) and LCF patients (median PFS, 24.8 months, p<0.001), while the OS of ECF patients (median OS, 13.0 months) was also significantly shorter than those in the control group (median OS, unreached, p<0.001) and LCF patients (median OS, 62.5 months, p<0.001, **Figure 2B**). It is noteworthy that the PFS (median PFS, 6.2 months) and OS (median OS, 13.0 months) of ECF patients were significantly shorter than those in the high-risk group according to the revised IPI score (median PFS, 24.5 months, p<0.001, median OS, 63.9 months, p<0.001). (**Supplementary Figures 2A-D**).

**Table 1:** Clinical and pathological characteristics of the patients in the control (n=1338) and ECF groups (n=376).

Characteristic	State		p value
	Control (n=1338)	ECF (n=376)	
Age≥60			0.000^*^
Yes	529/1338 (39.5%)	195/376 (51.9%)	
No	809/1338 (60.5%)	181/376 (48.1%)	
Ann Arbor stage			0.000^*^
I-II	845/1338 (63.2%)	97/376 (25.8%)	
III-IV	493/1338 (36.8%)	279/376 (74.2%)	
LDH			0.000^*^
Normal	880/1338 (65.8%)	104/376 (27.7%)	
Elevated	458/1338 (34.2%)	272/376 (72.3%)	
ECOG score			0.000^*^
0-1	1245/1338 (93.0%)	289/376 (76.9%)	
≥2	93/1338 (7.0%)	87/376 (23.1%)	
Extranodal involvements			0.000^*^
0-1	1100/1338 (82.2%)	224/376 (59.6%)	
≥2	238/1338 (17.8%)	152/376 (40.4%)	
Hans (n=1438)			0.022^*^
GCB	479/1141 (42.0%)	103/297 (34.7%)	
nonGCB	662/1141 (58.0%)	194/297 (65.3%)	
DEL (n=1042)			0.000^*^
Yes	172/866 (19.9%)	65/176 (36.9%)	
No	694/866 (80.1%)	111/176 (63.1%)	

a p value indicated the difference between DLBCL patients in the control and ECF group.

LDH, lactate dehydrogenase; ECOG, eastern cooperative oncology group; GCB, germinal center B-cell; DEL, double expression.

The symbol * means that the comparison between the two groups is statistically different, P<0.05.

### Mutational profile

Regarding the genetic profile, 55 genes related to the tumorigenesis of DLBCL were analyzed in 915 patients (control, n=616, ECF, n=148, and LCF, n=151, **Supplementary Figure 3**). The most frequently mutated genes (mutation frequency >15%) in the ECF group were *PIM1* (25.7%), *TP53* (21.6%), *BTG2* (21.0%), *KMT2D* (21.0%), *MYD88* (19.6%) and *CD79B* (16.2%). The mutation frequencies of *TP53* (21.6% vs. 11.2%, p=0.001), *FOXO1* (10.8% vs. 5.4%, p=0.015), *FBXW7* (4.1% vs. 1.3%, p=0.025) were significantly increased in the ECF group as compared to the control group (**Figure 2C**). The gene mutation burden of the ECF group was higher than the control group (**Figure 2D**, p=0.044). As for molecular subtype, the proportion of TP53^Mut^ subtypes in the ECF group was significantly higher than the control group (21.6% vs. 11.2%, p=0.001), and there was no statistically significant difference in the proportion of other subtypes between the two groups (**Figure 2E**).

As for LCF, the mutation frequencies of *MYD88* (29.1% % vs. 18.0%, p=0.002), *TP53* (18.5% vs. 11.2%, p=0.015), *EZH2* (10.6% vs. 6.0%, p=0.046), *CD79A* (4.6% vs. 1.8%, p=0.038) were significantly increased in the LCF group as compared to the control group (**Supplementary Figure 4A**). There was no statistically significant difference in the gene mutation burden between the LCF and control groups (**Supplementary Figure 4B**). Regarding the LymphPlex classification, the proportion of TP53^Mut^ subtypes (p=0.015) was significantly higher, while the proportion of BN2 subtypes (p=0.019) was markedly lower in the LCF group as compared to the control group (**Supplementary Figure 4C**). When compared with LCF, the mutation frequencies of *HIST1H1C* (6.8% % vs. 2.0%, p=0.044) and *LYN* (4.7% vs. 0.7%, p=0.030) were significantly increased in the ECF group. Similarly, the gene mutation burden (p=0.029) was also significantly higher in ECF **Supplementary Figures 5A, B**). No significant difference was observed in the molecular subtypes between LCF and ECF (**Supplementary Figure 5C**).

### Differential gene and pathway enrichment analysis

**Figure 3:**
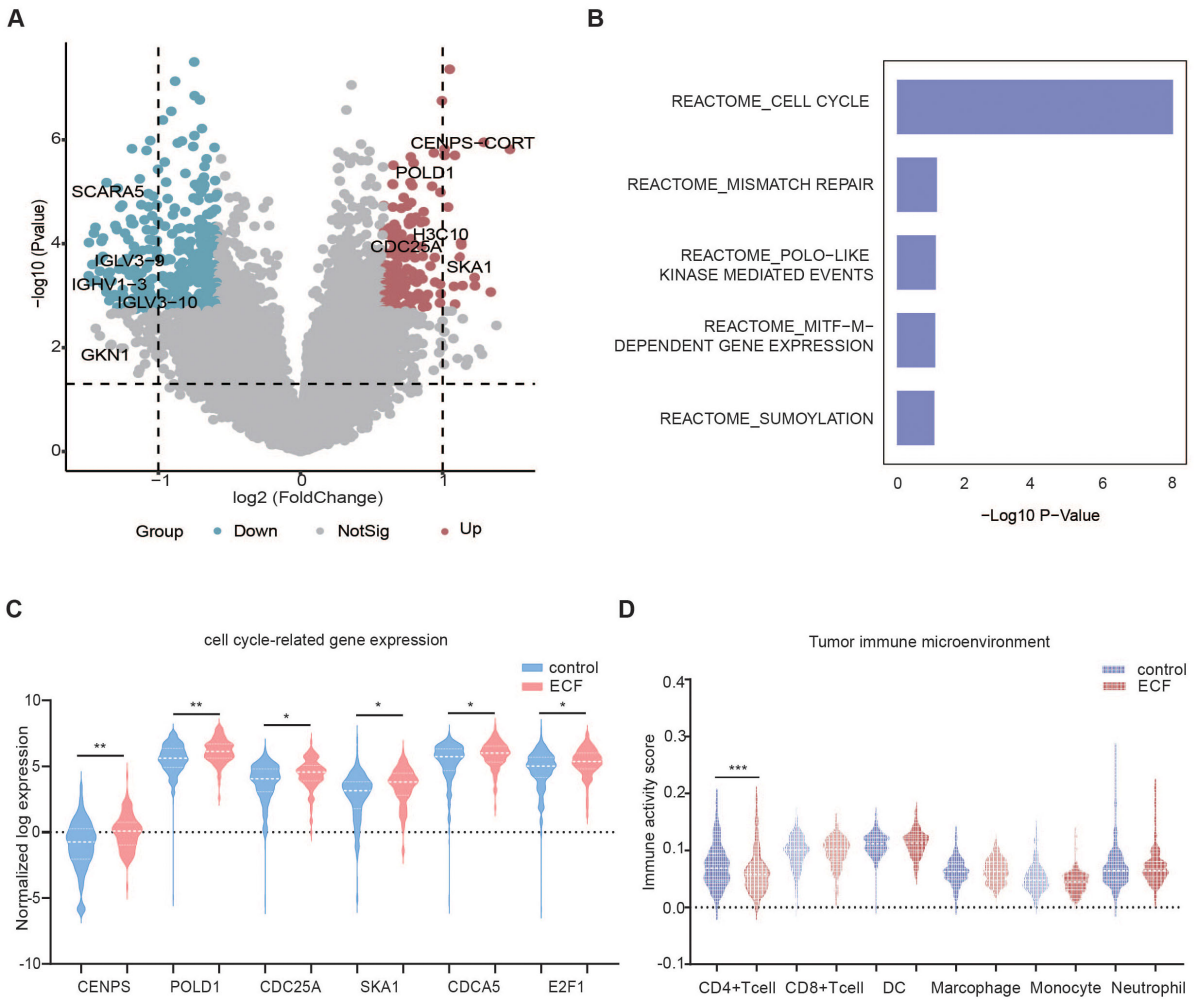
Differential gene expression and immune cell infiltration in the control and ECF groups. **(A)** The volcano plots show the differential expression of genes in the control (n=274) and ECF (n=97) groups. **(B)** Up-regulated pathways in ECF patients compared to the control group. **(C, D)** Violin plot of cell cycle-related genes and tumor microenvironment, cell cycle signaling-related genes **(C)** Tumor microenvironment in the control (n=274) and ECF (n=97) groups **(D)**. The symbols *, **, and *** indicate the levels of statistical significance for each variable. Specifically, * denotes a p-value less than 0.05, suggesting the result is statistically significant at the 5% level. ** indicates a p-value less than 0.01, showing stronger significance, while *** represents a p-value less than 0.001, implying a highly significant result.

RNA sequencing was performed on 474 lymphoma tissues, including 274 in the control group, 97 in the ECF group, and 103 in the LCF group. Compared to the control group, the ECF group differed significantly in gene expression pattern, with 530 genes differentially expressed. Of those, 183 genes were upregulated in the ECF group (**Figure 3A**). The GSEA analysis unveiled that the signaling pathways related to cell cycle, mismatch repair, polo-like kinase mediated events, Melanocyte Inducing Transcription Factor (MITF)-M-dependent gene expression and sumoylation were enhanced in ECF patients compared to the control group (**Figure 3B**). Among the genes related to the top 1 signaling pathway (cell cycle), the expression levels of *GENPS-CORT, POLD1, H3C10, CDC25A* and *SKA1* were notably increased in ECF (**Figure 3C**).

For LCF patients, a similar trend was observed in the upregulation of signaling pathways related to rRNA procession, Activated PKN1 stimulates transcription of androgen receptor (AR)-regulated genes KLK2 and KLK3, DNA replication, chromatin modifaction and rRNA expression according to the GSEA analysis (**Supplementary Figures 6A, B**).

Compared with the LCF group, the cell cycle checkpoints, rRNA procession, translation, DNA repair, processing of capped intron containing pre mRNA signaling pathways were upregulated in the ECF group (**Supplementary Figures 6C, D**).

### Tumor microenvironment of ECF

TME was evaluated by a web server ImmuCellAI using RNA sequencing data (35). According to the ImmuCellAI results, patients in the control group had significantly higher CD4^+^ T cell infiltration in TME (p=0.001) as compared to those in the ECF group (**Figure 3D**). For LCF patients, cytotoxic and exhausted T cells were significantly higher in TME than in the control group (**Supplementary Figure 6E**). However, when compared to the LCF group, the increased recruiting activity of neutrophils (p=0.038) and the decreased recruiting activity of CD4^+^ T cells (p=0.001) and CD8^+^ T cells (p=0.012) were observed in ECF patients (**Supplementary Figure 6F**).

### Construction and validation of the nomogram of ECF

Univariate and multivariate logistic regression analyses were performed to identify the potential risk factors for ECF patients. The univariate analysis was performed on 2038 patients, and revealed that elderly age (> 60 years), ECOG ≥2, Ann Arbor stage III/IV, elevated serum LDH level, multiple extranodal involvements, and mutations in *TP53*, *FOXO1*, and *FBXW7* genes were associated with increased risk of ECF (**Supplementary Table 3**). Multivariate logistic regression revealed that Age >60 (OR = 1.71, 95% [CI] = 1.11 ~ 2.63, P = 0.016), Ann Arbor stage III/IV (OR = 2.08, 95% [CI] = 1.23 ~ 3.52, P =0.006), elevated serum LDH level (OR = 3.63, 95% [CI] = 2.22 ~ 5.95, P <0.001), *TP53* mutation (OR = 1.91, 95% [CI] = 1.12 ~ 3.26; P =0.017) and *FOXO1* mutation (OR = 2.61, 95% [CI] = 1.24 ~ 5.47; P =0.011) were independent risk factors for ECF (**Supplementary Figure 7A**).

**Figure 4:**
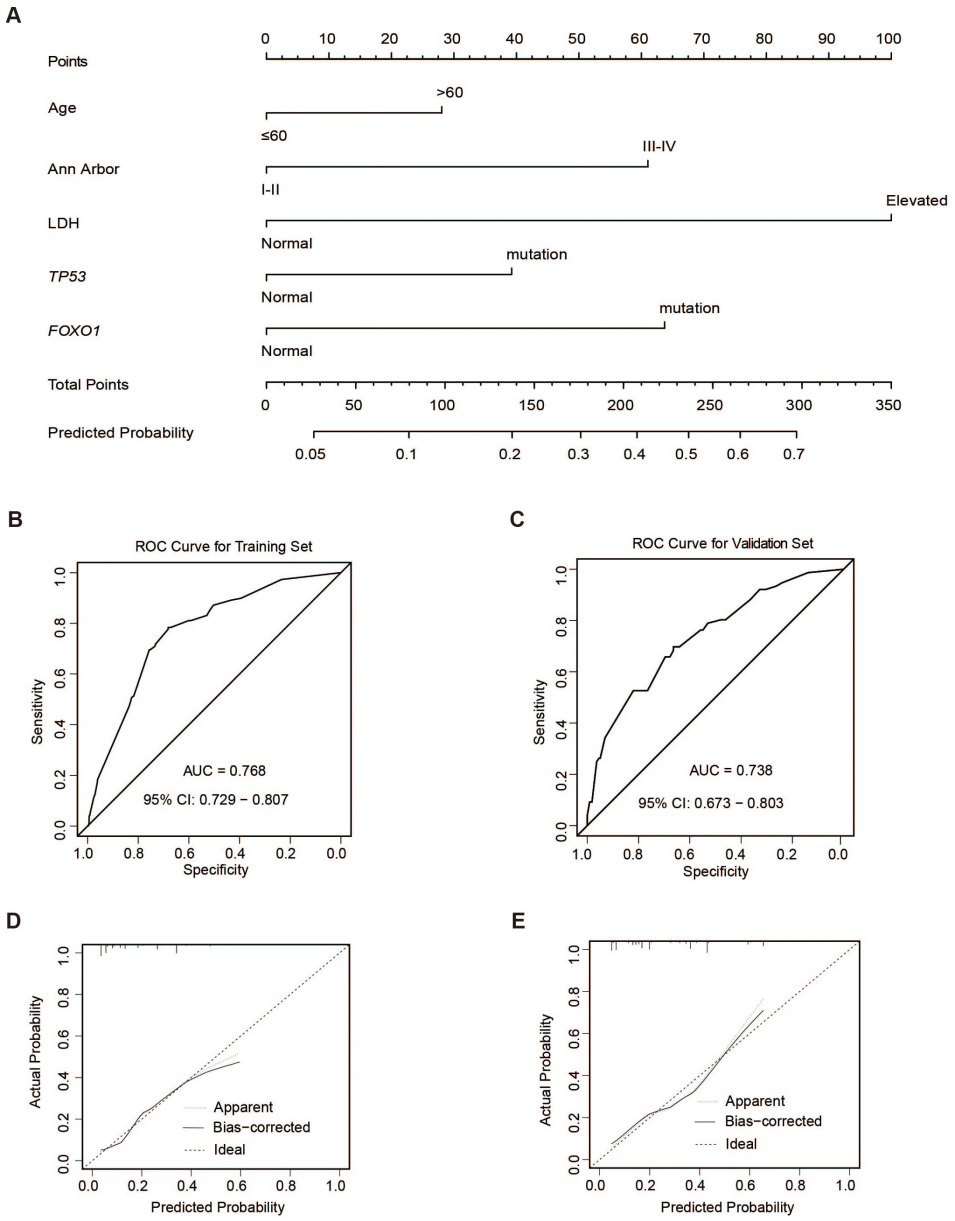
ECF nomogram. **(A)** Construction of ECF nomogram. **(B, C)** ROC curves of the training (n=2038) **(B)** and validation sets (BCC cohort n=320) **(C)**. **(D, E)** Calibration curves of the training **(D)** and validation sets **(E)**.

Based on these independent risk factors of ECF, we developed a nomogram to predict the risk of ECF (**Figure 4A**). 1000 bootstrap samples were used to test the performance of the prediction model. Through bootstrap analysis, the C-index of the model was 0.768 (**Figure 4B**). The calibration curve (**Figure 4D**) also showed good agreement between the predicted and actual outcomes. To further evaluate the accuracy and reliability of our nomogram, we performed external validation using BCC cohort from Western countries (36) and the results were satisfactory. Clinical and pathological characteristics of patients in the validation cohort were listed in **Supplementary Table 4**. The AUC value for the external validation of the BCC cohort (n = 320) was 0.738 (**Figure 4C**). The nomogram was found to fit well in both training and validation cohorts according to the Hosmer-Lemeshow test (**Figures 4D, E**). Overall, these findings indicated that the nomogram we constructed was a feasible and effective tool for predicting the risk of ECF patients.

## Discussion

ECF patients bear the dismal prognoses and are worthy of more attention (5, 7, 38, 39). To our knowledge, our study represents a relatively large cohort of Chinese DLBCL individuals with clinical and molecular characterizations, aiming to establish a predictive model capable of discerning ECF patients. This will provide clinicians with an efficient tool to expeditiously identify patients with the poorest survival outcomes, thus enabling the exploration of novel treatment approaches beyond R-CHOP. Multivariate analysis revealed that age >60, advanced Ann Arbor stage, elevated serum LDH, *TP53*, and *FOXO1* mutations were independent predictors of ECF, which enabled us to construct a nomogram to predict the risk of ECF. These results are highly consistent with the worse prognosis of TP53^Mut^ subtypes (40). Furthermore, mutated *TP53* inhibited the virus response and interferon release in DLBCL, and subsequently induced the suppressive TME with the lower infiltration of T-cells, contributing to the worse outcome of *TP53-*mutant patients under immune-targeted therapy (41). Of note, Rushton and colleagues found that these mutations in *the TP53* gene remained clonally persistent throughout treatment, explaining its role in primary treatment resistance and its resistance to subsequent high-dose chemotherapy (42) and CAR-T treatment (43). Therefore, *TP53* mutations facilitate the identification of potential ECF patients and have important value in the design of future therapeutic strategies. *FOXO1* is an important transcription factor, modulating the transcription of CD20. *FOXO1* mutation reduces the expression level of CD20, resulting in resistance to anti-CD20 therapy, and is associated with poor prognosis of patients treated with R-CHOP regimen (44–46). Noteworthy, the training cohort of Chinese patients and the external cohort from Western countries showed a good predictive power of ECF, regardless of patients’ race. As for clinical features, ECF patients had high proportions of elderly age, advanced Ann Arbor stage, and elevated serum LDH, indicating that the high IPI score is a strong predictor of drug resistance (9, 40). For molecular characteristics, the mutation rates of *TP53, FOXO1*, and *FBXW7* genes were high in the ECF group. In addition to *TP53* and *FOXO1*, *FBXW7*, an E3 ubiquitin-protein ligase, is a notch signaling suppressor. Mutation or loss of function may lead to abnormal activation of the notch signaling pathway, which further increases the expression of *CCL2* and *CSF1* and promotes the transformation of tumor-associated macrophages (TAM) to M2 phenotype, thus promoting lymphoma cell proliferation (29, 47).

Regarding the TME, our results showed that the infiltration of CD4+ T cells was significantly lower in the ECF group compared to the control group, while the recruitment activity of neutrophils was higher in the ECF group as compared to the LCF group. Our results were consistent with the observations that patients with high CD4+ T cell infiltration had better survival as compared to those with low CD4+ T cell infiltration (48). Cell-mediated immunity plays a key role in controlling tumor growth and progression in DLBCL (49–52). CD4+ T cells, by activating other immune cells in the TME, are associated with a favorable prognosis in lymphoma (53). In DLBCL, neutrophils are reported to form extracellular traps, then up-regulate the Toll-like receptor 9 pathway and subsequently promote the lymphoma progression (54). Therefore, the low infiltration of CD4+ T cells and the high recruitment of neutrophils formed the immunosuppressive TME of ECF patients. Exhausted T cells were significantly increased in the LCF group, which was associated with poor prognosis of patients (55).

In conclusion, our study demonstrated the clinical and molecular profiles of ECF and LCF patients and established an ECF nomogram, which paves the way for convenient identification of patients with a high risk of ECF, thereby allowing for the development of customized therapeutic strategies beyond the conventional R-CHOP treatment in DLBCL.

## Data Availability

The datasets presented in this study can be found in online repositories. The names of the repository/repositories and accession number(s) can be found below: https://www.biosino.org/node, OEP001143.

## Supplementary material

Supplementary Figure 1 Survival of DLBCL patients with SD/PD or relapsed within 1 year. **(A, B)** PFS **(A)** and OS **(B)** in patients with SD/PD to R-CHOP regimen (n=321) and those achieved CR/PR but relapsed within 1 year (n=81).

Supplementary Figure 2 Survival of DLBCL patients according to different risk models. (A-B) PFS **(A)** and OS **(B)** of patients with low-risk (n=1436) or high-risk (n=602). **(C-D)** PFS **(C)** and OS **(D)** of patients with high-risk (n=602), ECF (n=376) and LCF (n=324).

Supplementary Figure 3 Mutation profiles of DLBCL patients.

Supplementary Figure 4 Gene mutations of DLBCL patients in the control and LCF groups. **(A-C)** Mutation rates **(A)**, gene mutation burden **(B)**, and molecular subtypes **(C)** of patients in the control (n=616) and LCF (n=151) groups.

Supplementary Figure 5 Gene mutations of DLBCL patients in the ECF and LCF groups. **(A-C)** Mutation rates **(A)**, gene mutation burden **(B)**, and molecular subtypes **(C)** of patients in the ECF (n=148) and LCF (n=151) groups.

Supplementary Figure 6 Differential gene expressions and immune cell infiltration in the control VS LCF groups and LCF VS ECF groups. **(A)** The volcano plots show the differential expression of genes in the control (n=274) and LCF (n=103) group. **(B)** Up-regulated pathways in the LCF patients compared to the control group. **(C)** The volcano plots show the differential expression of genes in the ECF (n=97) and LCF groups (n=103). **(D)** Up-regulated pathways in the ECF patients compared to the LCF patients. **(E)**Immune cell infiltrations in the control (n=274) and LCF (n=103) groups. **(F)** Immune cell infiltrations in the ECF (n=97) and LCF groups (n=103).

Supplementary Figure 7 Multivariate analysis of ECF.
