# Supplementary figures and images for: Characteristics and predictive model for diffuse large B-cell lymphoma with early chemoimmunotherapy failure
Source: Front Immunol. 2025 Jun 16;16:1553850. doi: 10.3389/fimmu.2025.1553850 (PMC12206878; doi:10.3389/fimmu.2025.1553850)

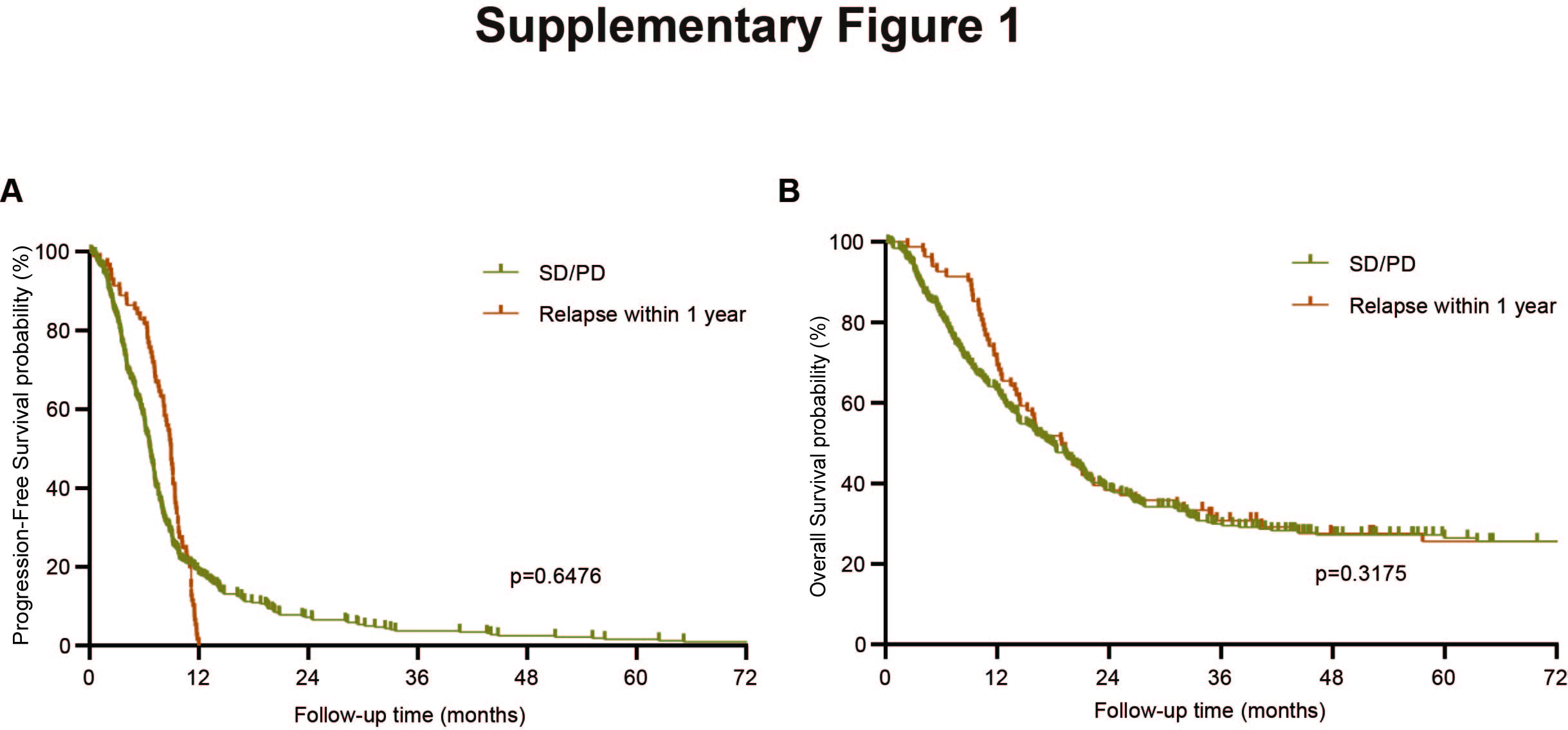

Supplement: Supplementary Figure 1 — Survival of DLBCL patients with SD/PD or relapsed within 1 year. (A, B) PFS (A) and OS (B) in patients with SD/PD to R-CHOP regimen (n=321) and those achieved CR/PR but relapsed within 1 year (n=81). [file Image1.jpeg]

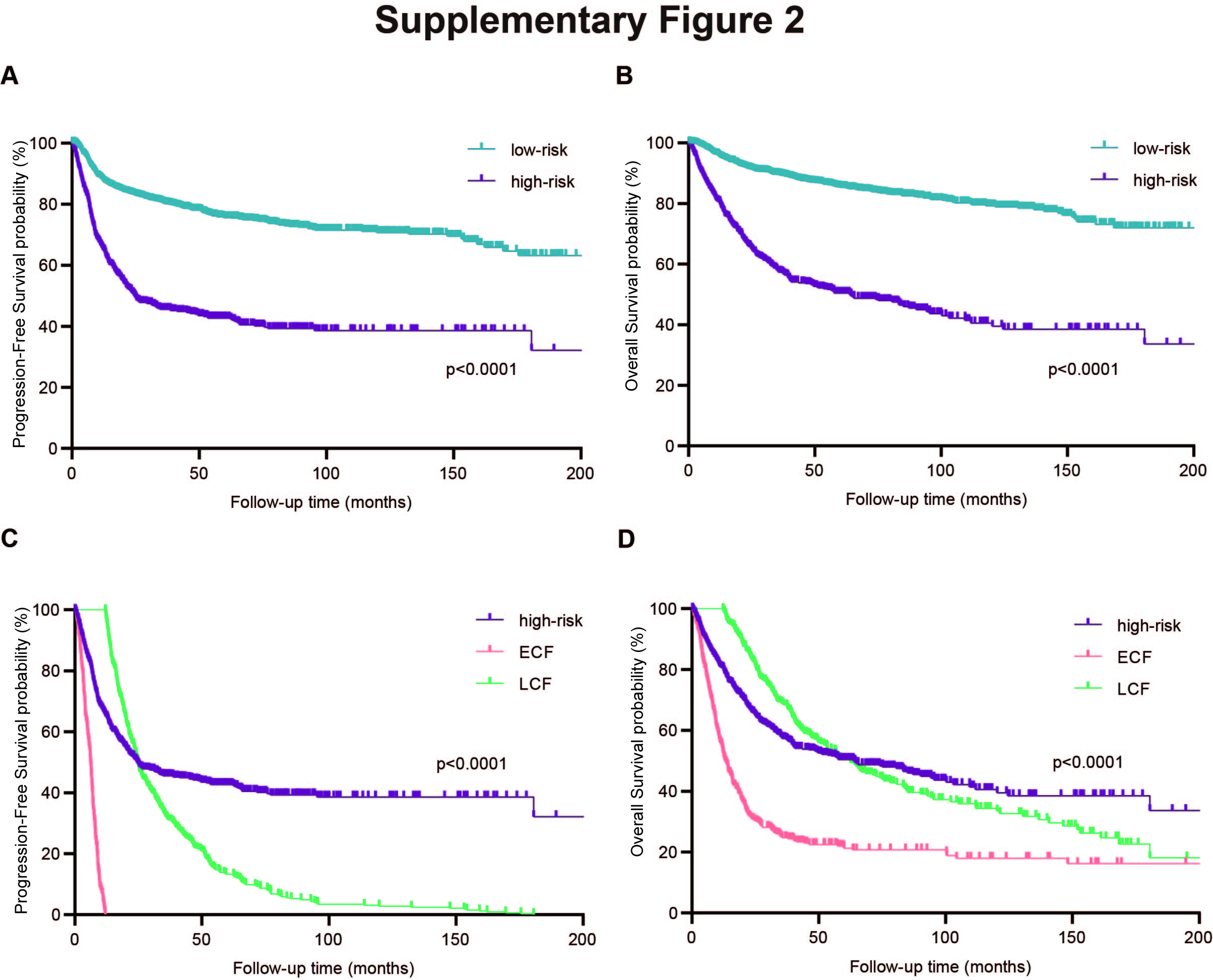

Supplement: Supplementary Figure 2 — Survival of DLBCL patients according to different risk models. (A-B) PFS (A) and OS (B) of patients with low-risk (n=1436) or high-risk (n=602). (C-D) PFS (C) and OS (D) of patients with high-risk (n=602), ECF (n=376) and LCF (n=324). [file Image2.jpeg]

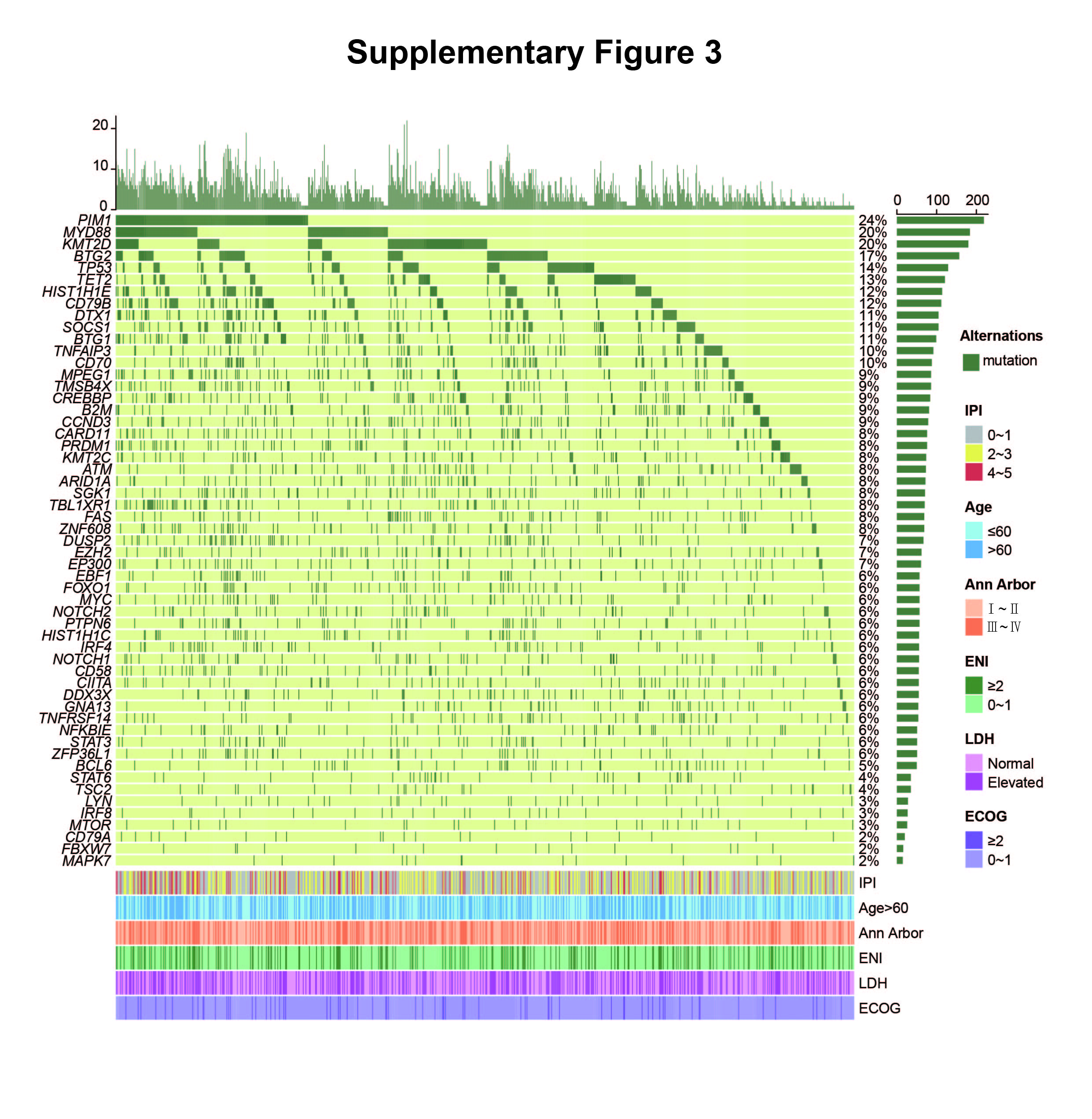

Supplement: Supplementary Figure 3 — Mutation profiles of DLBCL patients. [file Image3.jpeg]

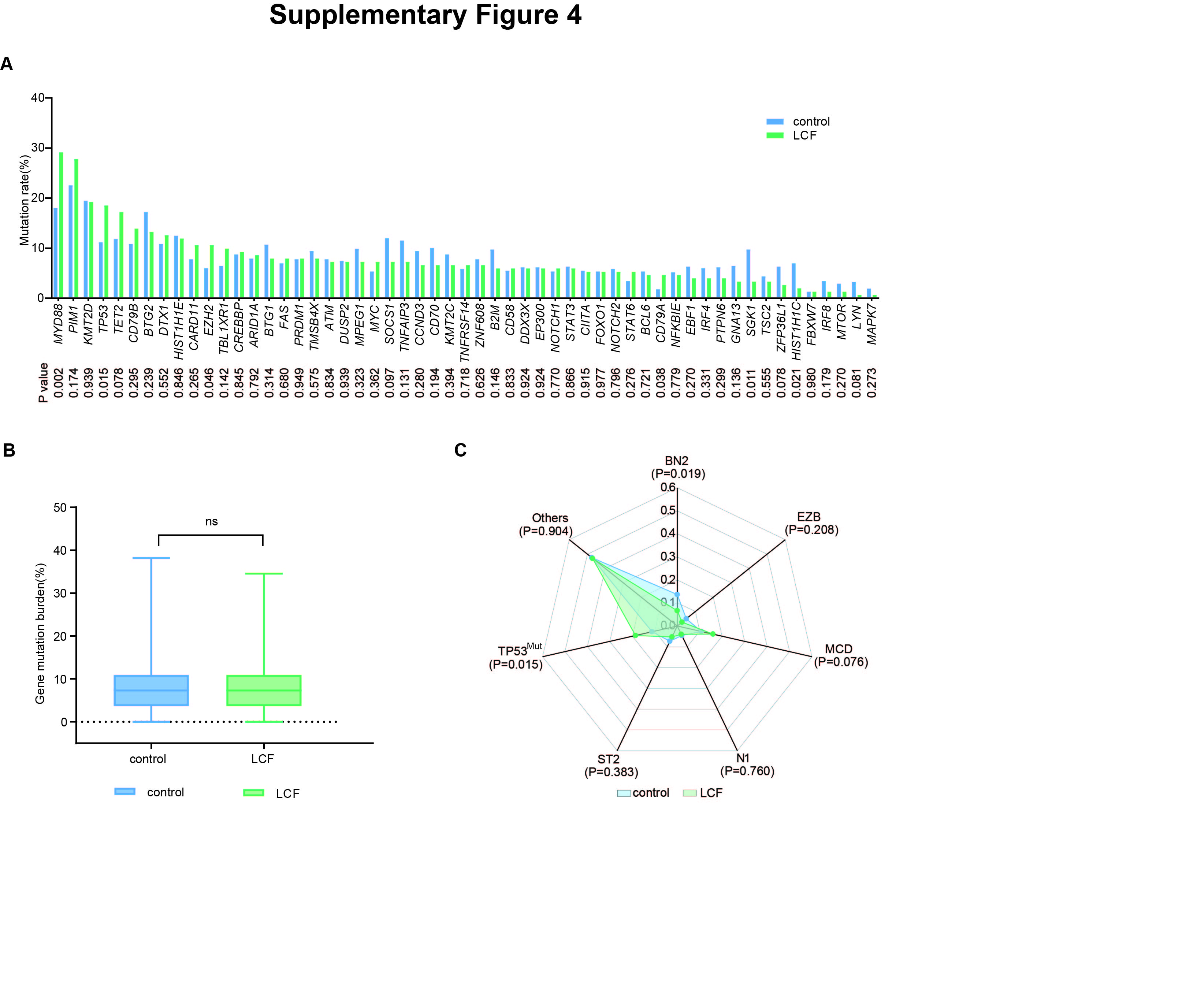

Supplement: Supplementary Figure 4 — Gene mutations of DLBCL patients in the control and LCF groups. (A-C) Mutation rates (A), gene mutation burden (B), and molecular subtypes (C) of patients in the control (n=616) and LCF (n=151) groups. [file Image4.jpeg]

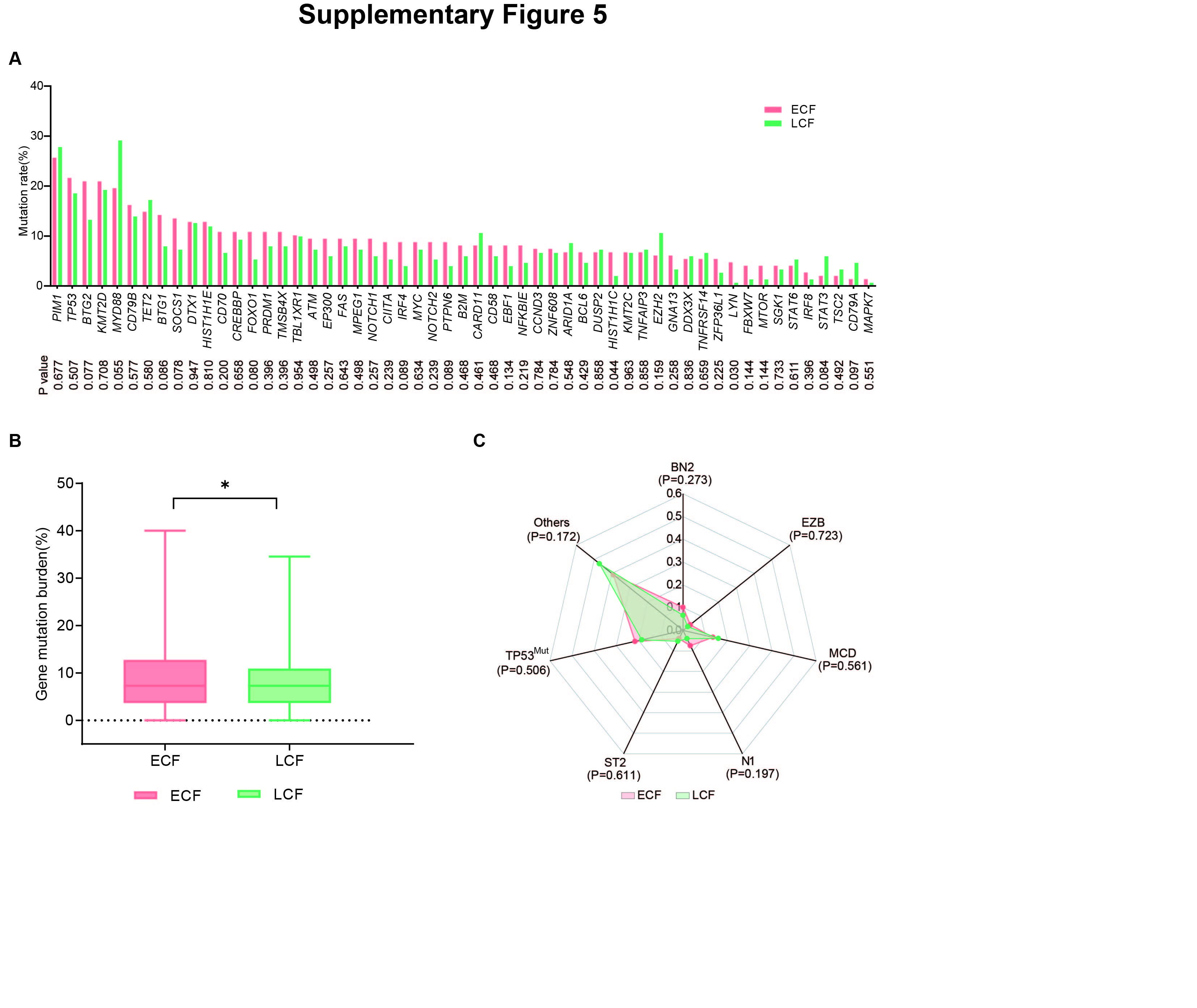

Supplement: Supplementary Figure 5 — Gene mutations of DLBCL patients in the ECF and LCF groups. (A-C) Mutation rates (A), gene mutation burden (B), and molecular subtypes (C) of patients in the ECF (n=148) and LCF (n=151) groups. [file Image5.jpeg]

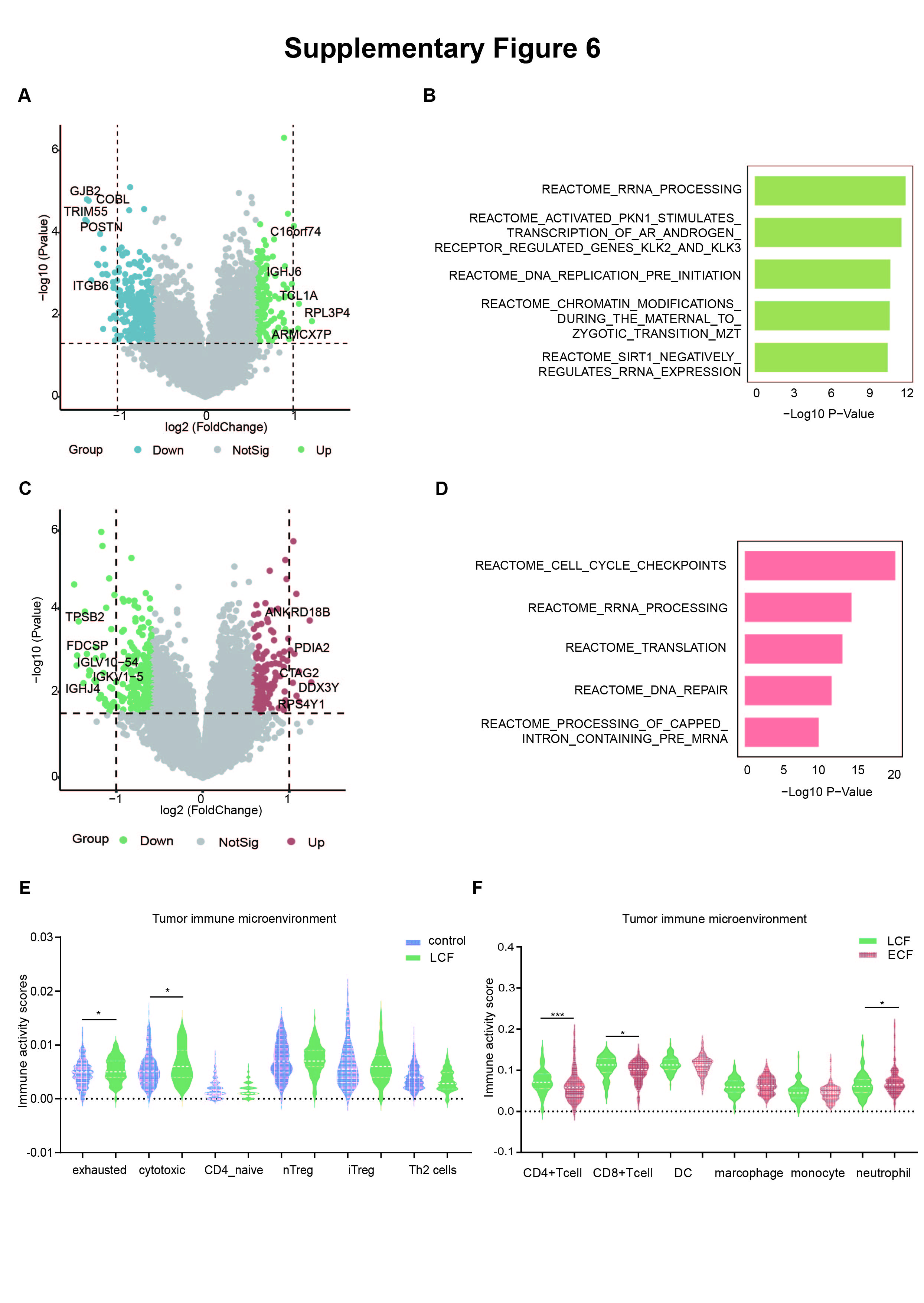

Supplement: Supplementary Figure 6 — Differential gene expressions and immune cell infiltration in the control VS LCF groups and LCF VS ECF groups. (A) The volcano plots show the differential expression of genes in the control (n=274) and LCF (n=103) group. (B) Up-regulated pathways in the LCF patients compared to the control group. (C) The volcano plots show the differential expression of genes in the ECF (n=97) and LCF groups (n=103). (D) Up-regulated pathways in the ECF patients compared to the LCF patients. (E)Immune cell infiltrations in the control (n=274) and LCF (n=103) groups. (F) Immune cell infiltrations in the ECF (n=97) and LCF groups (n=103). [file Image6.jpeg]

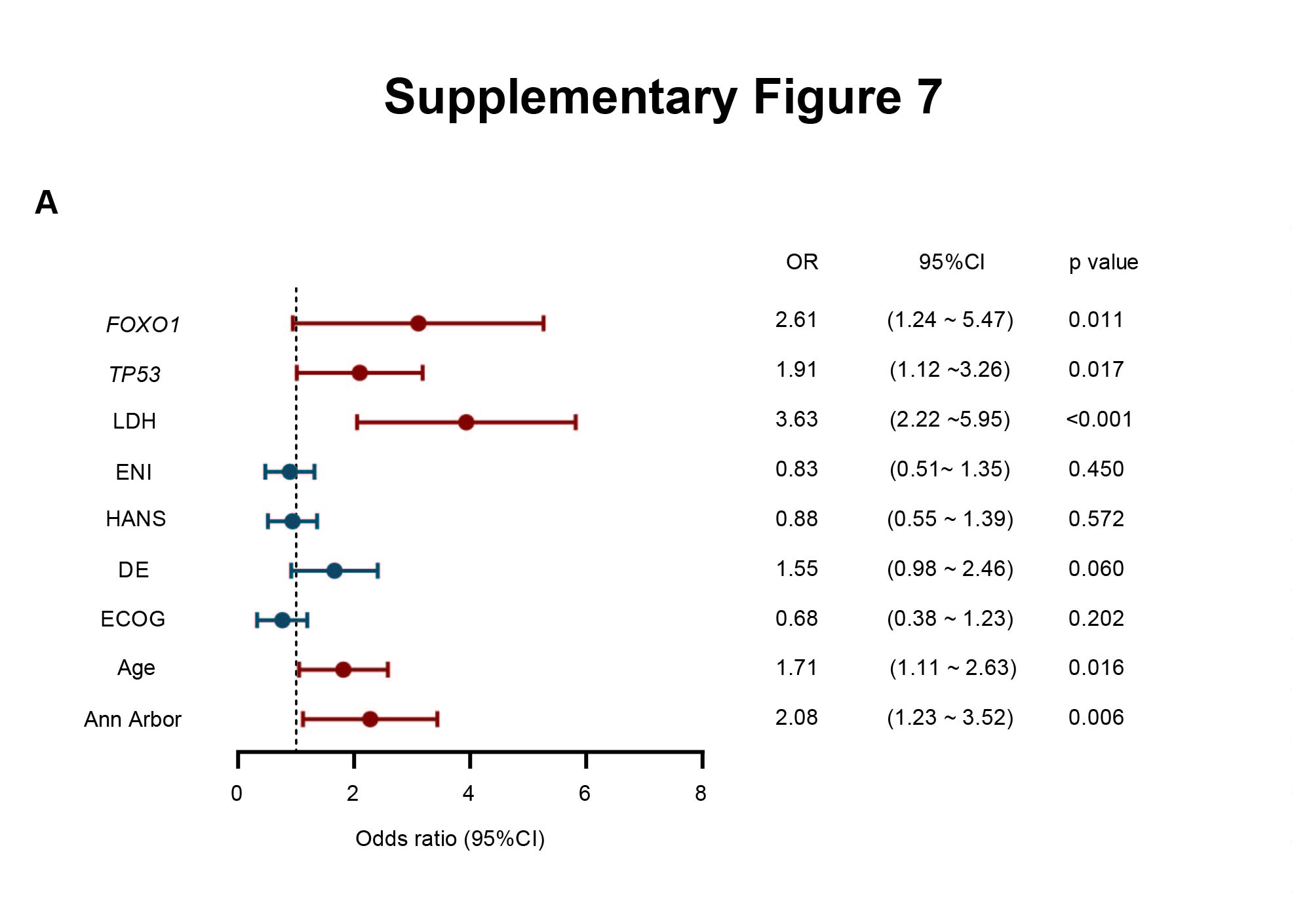

Supplement: Supplementary Figure 7 — Multivariate analysis of ECF. [file Image7.jpeg]
