# Supplementary material for: Characteristics and predictive model for diffuse large B-cell lymphoma with early chemoimmunotherapy failure
Source: Front Immunol. 2025 Jun 16;16:1553850. doi: 10.3389/fimmu.2025.1553850 (PMC12206878; doi:10.3389/fimmu.2025.1553850)
Supplement: Supplementary file 8 [file Table1.docx]

**Supplementary Table 1. Clinical and pathological characteristics of patients in the control and LCF groups**

| Characteristic | State | | | | p value |
| --- | --- | --- | --- | --- | --- |
|  | control (n=1338) | | | LCF (n=324) |  |
| Age≥60 |  | | |  | 0.000^*^ |
| Yes | 529/1338 (39.5%) | | | 166/324 (51.2%) |  |
| No | 809/1338 (60.5%) | | | 158/324 (48.8%) |  |
| Ann Arbor stage |  | | |  | 0.000^*^ |
| Ⅰ-Ⅱ | 845/1338 (63.2%) | | | 121/324 (37.3%) |  |
| Ⅲ-Ⅳ | 493/1338 (36.8%) | | | 203/324 (62.7%) |  |
| LDH |  | | |  | 0.000^*^ |
| Normal | 880/1338 (65.8%) | | | 150/324 (46.3%) |  |
| Elevated | 458/1338 (34.2%) | | | 174/324 (53.7%) |  |
| ECOG score |  | | |  | 0.002^*^ |
| 0-1 | 1245/1338 (93.0%) | | | 285/324 (88.0%) |  |
| ≥2 | 93/1338 (7.0%) | | | 39/324 (12.0%) |  |
| Extranodal involvements |  | |  | | 0.000^*^ |
| 0-1 | 1100/1338 (82.2%) | | | 212/324 (65.4%) |  |
| ≥2 | 238/1338 (17.8%) | | | 112/324 (34.6%) |  |
| Hans (n=1399) |  | | |  | 0.000^*^ |
| GCB | 479/1141 (42.0%) | | | 72/258 (27.9%) |  |
| nonGCB | 662/1141 (58.0%) | | | 186/258 (72.1%) |  |
| DEL (n=1050) | |  | |  | 0.041^*^ |
| Yes | 172/866 (19.9%) | | | 49/184 (26.6%) |  |
| No | 694/866 (80.1%) | | | 135/184 (73.4%) |  |

**Supplementary Table 2. Clinical and pathological characteristics of patients in the ECF and LCF groups**

| Characteristic | State | | | p value |
| --- | --- | --- | --- | --- |
|  | ECF (n=376) | | LCF (n=324) |  |
| Age≥60 |  | |  | 0.869 |
| Yes | 195/376 (51.9%) | | 166/324 (51.2%) |  |
| No | 181/376 (48.1%) | | 158/324 (48.8%) |  |
| Ann Arbor stage |  | |  | 0.001^*^ |
| Ⅰ-Ⅱ | 97/376 (25.8%) | | 121/324 (37.3%) |  |
| Ⅲ-Ⅳ | 279/376 (74.2%) | | 203/324 (62.7%) |  |
| LDH |  | |  | 0.000^*^ |
| Normal | 104/376 (27.7%) | | 150/324 (46.3%) |  |
| Elevated | 272/376 (72.3%) | | 174/324 (53.7%) |  |
| ECOG score |  | |  | 0.000^*^ |
| 0-1 | 289/376 (76.9%) | | 285/324 (88.0%) |  |
| ≥2 | 87/376 (23.1%) | | 39/324 (12.0%) |  |
| Extranodal involvements | |  |  | 0.111 |
| 0-1 | 224/376 (59.6%) | | 212/324 (65.4%) |  |
| ≥2 | 152/376 (40.4%) | | 112/324 (34.6%) |  |
| Hans (n=555) |  | |  | 0.087 |
| GCB | 103/297 (34.7%) | | 72/258 (27.9%) |  |
| nonGCB | 194/297 (65.3%) | | 186/258 (72.1%) |  |
| DEL (n=360) | |  |  | 0.036^*^ |
| Yes | 65/176 (36.9%) | | 49/184 (26.6%) |  |
| No | 111/176 (63.1%) | | 135/184 (73.4%) |  |

**Supplementary Table 3. Univariate and multivariate analysis of the predictors for ECF**

| Variables |  | Univariate | | | | |  | Multivariate | | | | |
| --- | --- | --- | --- | --- | --- | --- | --- | --- | --- | --- | --- | --- |
|  |  | β | S. E | Z | p | OR (95%CI) |  | β | S. E | Z | p | OR (95%CI) |
| Ann Arbor stage | | | |  |  |  |  |  |  |  |  |  |
| Ⅰ-Ⅱ |  |  |  |  |  | 1.00 (Reference) |  |  |  |  |  | 1.00 (Reference) |
| Ⅲ-Ⅳ |  | 1.38 | 0.13 | 10.82 | <.001 | 3.99 (3.11 ~ 5.13) |  | 0.73 | 0.27 | 2.74 | 0.006 | 2.08 (1.23 ~ 3.52) |
| ECOG score |  |  |  |  |  |  |  |  |  |  |  |  |
| ≥2 |  |  |  |  |  | 1.00 (Reference) |  |  |  |  |  | 1.00 (Reference) |
| 0~1 |  | -1.25 | 0.15 | -8.21 | <.001 | 0.29 (0.21 ~ 0.39) |  | -0.39 | 0.30 | -1.28 | 0.202 | 0.68 (0.38 ~ 1.23) |
| Extranodal involvements | | | | |  |  |  |  |  |  |  |  |
| ≥2 |  |  |  |  |  | 1.00 (Reference) |  |  |  |  |  | 1.00 (Reference) |
| 0~1 |  | -0.93 | 0.12 | -7.71 | <.001 | 0.39 (0.31 ~ 0.50) |  | -0.19 | 0.25 | -0.75 | 0.450 | 0.83 (0.51 ~ 1.35) |
| Hans |  |  |  |  |  |  |  |  |  |  |  |  |
| GCB |  |  |  |  |  | 1.00 (Reference) |  |  |  |  |  | 1.00 (Reference) |
| nonGCB |  | 0.20 | 0.13 | 1.51 | 0.131 | 1.22 (0.94 ~ 1.59) |  | -0.13 | 0.23 | -0.56 | 0.572 | 0.88 (0.55 ~ 1.39) |
| Age>60 |  |  |  |  |  |  |  |  |  |  |  |  |
| No |  |  |  |  |  | 1.00 (Reference) |  |  |  |  |  | 1.00 (Reference) |
| Yes |  | 0.40 | 0.11 | 3.53 | <.001 | 1.50 (1.20 ~ 1.88) |  | 0.53 | 0.22 | 2.42 | 0.016 | 1.71 (1.11 ~ 2.63) |
| LDH |  |  |  |  |  |  |  |  |  |  |  |  |
| Normal |  |  |  |  |  | 1.00 (Reference) |  |  |  |  |  | 1.00 (Reference) |
| Elevated |  | 1.45 | 0.13 | 11.52 | <.001 | 4.26 (3.33 ~ 5.46) |  | 1.29 | 0.25 | 5.12 | <.001 | 3.63 (2.22 ~ 5.95) |
| DEL |  |  |  |  |  |  |  |  |  |  |  |  |
| No |  |  |  |  |  | 1.00 (Reference) |  |  |  |  |  | 1.00 (Reference) |
| Yes |  | 0.79 | 0.17 | 4.53 | <.001 | 2.20 (1.56 ~ 3.09) |  | 0.44 | 0.23 | 1.88 | 0.060 | 1.55 (0.98 ~ 2.46) |
| *TP53* |  |  |  |  |  |  |  |  |  |  |  |  |
| unmutation |  |  |  |  |  | 1.00 (Reference) |  |  |  |  |  | 1.00 (Reference) |
| mutation |  | 0.64 | 0.23 | 2.84 | 0.005 | 1.91 (1.22 ~ 2.97) |  | 0.65 | 0.27 | 2.38 | 0.017 | 1.91 (1.12 ~ 3.26) |
| *FBXW7* |  |  |  |  |  |  |  |  |  |  |  |  |
| unmutation |  |  |  |  |  | 1.00 (Reference) |  |  |  |  |  | 1.00 (Reference) |
| mutation |  | 1.16 | 0.52 | 2.22 | 0.027 | 3.20 (1.14 ~ 8.94) |  | 0.70 | 0.66 | 1.06 | 0.290 | 2.02 (0.55 ~ 7.43) |
| *FOXO1* |  |  |  |  |  |  |  |  |  |  |  |  |
| unmutation |  |  |  |  |  | 1.00 (Reference) |  |  |  |  |  | 1.00 (Reference) |
| mutation |  | 0.76 | 0.31 | 2.47 | 0.014 | 2.15 (1.17 ~ 3.94) |  | 0.96 | 0.38 | 2.54 | 0.011 | 2.61 (1.24 ~ 5.47) |

**Supplementary Table 4. Clinical and pathological characteristics of patients in the validation cohort**

| Characteristic | State | | | |  | p value |
| --- | --- | --- | --- | --- | --- | --- |
|  | control (n=160) | ECF (n=82) | | | LCF (n=78) |  |
| Age≥60 |  |  | | |  | 0.000^*^ |
| Yes | 74/160 (46.3%) | 54/82 (65.9%) | | | 61/78 (78.2%) |  |
| No | 86/160 (53.7%) | 28/82 (34.1%) | | | 17/78 (21.8%) |  |
| Ann Arbor stage (n=317) | |  | | |  | 0.000^*^ |
| Ⅰ-Ⅱ | 99/160 (61.9%) | 20/81 (24.7%) | | | 33/76 (43.4%) |  |
| Ⅲ-Ⅳ | 61/160 (38.1%) | 61/81 (75.3%) | | | 43/76 (56.6%) |  |
| LDH (n=293) |  |  | | |  | 0.000^*^ |
| Normal | 90/148 (60.8%) | 18/76 (23.7%) | | | 30/69 (43.5%) |  |
| Elevated | 58/148 (39.2%) | 58/76 (76.3%) | | | 39/69 (56.5%) |  |
| ECOG score (n=317) |  |  | | |  | 0.000^*^ |
| 0-1 | 122/160 (76.3%) | 37/81 (45.7%) | | | 56/76 (73.7%) |  |
| ≥2 | 38/160 (23.7%) | 44/81 (54.3%) | | | 20/76 (26.3%) |  |
| Extranodal involvements (n=317) | | |  | |  | 0.147 |
| 0-1 | 143/160 (89.4%) | 65/81 (80.2%) | | | 66/76 (86.8%) |  |
| ≥2 | 17/160 (10.6%) | 16/81 (19.8%) | | | 10/76 (13.2%) |  |
| Hans (n=315) |  |  | | |  | 0.027^*^ |
| GCB | 107/158 (67.7%) | 37/80 (46.3%) | | | 44/77 (57.1%) |  |
| nonGCB | 51/158 (32.3%) | 43/80(53.7%) | | | 33/77 (42.9%) |  |
| DEL (n=313) |  | | |  |  | 0.003^*^ |
| Yes | 37/156 (23.7%) | 36/79 (45.6%) | | | 24/78 (30.8%) |  |
| No | 119/156 (76.3%) | 43/79 (54.4%) | | | 54/78 (69.2%) |  |

**Supplementary Table 1. Clinical and pathological characteristics of the patients in the control (n=1338) and LCF groups (n=324)**

^a^p value indicated the difference between DLBCL patients in the control and LCF groups.

Abbreviations: LDH, lactate dehydrogenase; ECOG, eastern cooperative oncology group; GCB, germinal center B-cell; DEL, double expression.

**Supplementary Table 2.** **Clinical and pathological characteristics of the patients in the ECF (n=376) and LCF groups (n=324)**

^a^p value indicated the difference between DLBCL patients in the ECF and LCF groups.

Abbreviations: LDH, lactate dehydrogenase; ECOG, eastern cooperative oncology group; GCB, germinal center B-cell; DEL, double expression.

**Supplementary Table 3. Univariate and multivariate analysis by Logistic regression** Abbreviations: ECOG, eastern cooperative oncology group; GCB, germinal center B-cell; LDH, lactate dehydrogenase; DEL, double expression.

**Supplementary Table 4. Clinical and pathological characteristics of patients in the validation cohort**

^a^p value indicated the difference between Validation cohort. Abbreviations: LDH, lactate dehydrogenase; ECOG, eastern cooperative oncology group; GCB, germinal center B-cell; DEL, double expression.
